# An Updated Overview of Metabolomic Profile Changes in Chronic Obstructive Pulmonary Disease

**DOI:** 10.3390/metabo9060111

**Published:** 2019-06-10

**Authors:** Nan Ran, Zhiqiang Pang, Yinuo Gu, He Pan, Xu Zuo, Xuewa Guan, Yuze Yuan, Ziyan Wang, Yingqiao Guo, Zixu Cui, Fang Wang

**Affiliations:** 1 Department of Pathogen Biology, College of Basic Medical Sciences, Jilin University, Changchun 130021, China; rannan17@mails.jlu.edu.cn (N.R.); pangzq2812@mails.jlu.edu.cn (Z.P.); guyn18@mails.jlu.edu.cn (Y.G.); panhe18@mails.jlu.edu.cn (H.P.); zuoxu18@mails.jlu.edu.cn (X.Z.); guanxw15@mails.jlu.edu.cn (X.G.); yuanyz17@mails.jlu.edu.cn (Y.Y.); wzy16@mails.jlu.edu.cn (Z.W.); 2 Department of Genetics, College of Basic Medical Sciences, Jilin University, Changchun 130021, China; yqguo16@mails.jlu.edu.cn (Y.G.); cuizx18@mails.jlu.edu.cn (Z.C.)

**Keywords:** metabolomics, COPD, inflammation, pathogenesis, biomarker

## Abstract

Chronic obstructive pulmonary disease (COPD), a common and heterogeneous respiratory disease, is characterized by persistent and incompletely reversible airflow limitation. Metabolomics is applied to analyze the difference of metabolic profile based on the low-molecular-weight metabolites (<1 kDa). Emerging metabolomic analysis may provide insights into the pathogenesis and diagnosis of COPD. This review aims to summarize the alteration of metabolites in blood/serum/plasma, urine, exhaled breath condensate, lung tissue samples, etc. from COPD individuals, thereby uncovering the potential pathogenesis of COPD according to the perturbed metabolic pathways. Metabolomic researches have indicated that the dysfunctions of amino acid metabolism, lipid metabolism, energy production pathways, and the imbalance of oxidations and antioxidations might lead to local and systematic inflammation by activating the Nuclear factor kappa-light-chain-enhancer of activated B cells signaling pathway and releasing inflammatory cytokines, like interleutin-6 (IL-6), tumor necrosis factor-α, and IL-8. In addition, they might cause protein malnutrition and oxidative stress and contribute to the development and exacerbation of COPD.

## 1. Introduction

Chronic obstructive pulmonary disease (COPD), characterized by persistent airflow limitation and incompletely reversible airway construction, will become the third leading cause of death worldwide by 2030 [1]. It is extremely important for COPD patients to receive comprehensive and personalized diagnosis and treatment strategies. Traditional diagnostic methods like pulmonary function tests, radiology, and bronchoscopy need comprehensive multiparameter analysis, and not every patient is suitable for them. Forced expiratory volume in 1 s, a validated clinical marker of COPD, has a poor correlation with clinical features and is not sensitive enough to predict the early onset of disease. Moreover, the great heterogeneity and phenotypic complexity of COPD may limit the accurate diagnosis and therapeutic effect evaluation in clinical phenomenon. Metabolomics, as a minimally invasive and sensitive approach, has increasingly been used in COPD-associated studies based on clinical patients and experimental animals [2,3]. A comprehensive understanding on metabolomic data may provide insights into the diagnosis, prognosis, and pharmacodynamic evaluation of COPD. In addition, these discriminating metabolites are also promising signatures for exploring the pathogenesis of COPD. Several metabolomics studies have shown that the alteration of sphingolipid metabolism was significantly related to the occurrence of COPD [4,5]. Currently, however, the confirmed special biomarkers associated with COPD have not been reported. The detection of metabolomic profile in COPD may help diagnose early patients and provide advisable potential therapeutic strategies for achieving the ultimate goal of precision medicine.

Previous metabolomic studies have mainly focused on the identification of differential biomarkers for the diagnosis of COPD [6]. Recently, researchers have taken the pathogenesis of COPD into consideration by integrating the metabolic pathways and cell signal pathways [7]. In this review, we summarize the changes of metabolites in COPD. Metabolic pathway analysis was performed by Metabo-Analyst 4.0 based on distinct metabolites identified in COPD-associated metabolomics studies. These different metabolites and disturbed metabolic pathways might contribute to uncovering the potential pathogenesis of COPD. We anticipate that this review can provide a whole overview for readers to metabolomic studies of COPD and a research basis for further exploring COPD-associated biomarkers and COPD pathogenesis.

## 2. COPD-Associated Biomarkers in Different Sample Types and Metabolic Pathway Analysis

Currently, high-throughput metabolomics studies have covered phenotypic differentiation, diagnosis, differential diagnosis, and therapeutic intervention, including pulmonary rehabilitation therapy for COPD [4,8,9,10,11]. Various samples including blood/serum/plasma, urine, exhaled breath condensate (EBC), lung tissue, sputum, bronchoalveolar lavage fluid (BALF), etc. [12] have been used. The distinct metabolites and disturbed metabolic pathways might provide evidence to illustrate the pathogenesis of COPD [13]. Distinct metabolites are shown in Table 1, which might provide a visual and comprehensive understanding for readers about COPD-associated metabolomics research in recent years. Moreover, the data shown in Table 1 might contribute to the exploration of COPD-associated biomarkers and potential COPD pathogenesis.

### 2.1. Blood/Serum/Plasma Sample

Compositions in blood/serum/plasma samples might be changed under local and systemic dysfunctions and pathological states. Therefore, these samples have been extensively used for metabolomics studies [14]. In particular, they have large potential for clinical diagnosis.

#### 2.1.1. Phenotypic Differentiation and Therapy of COPD

Lipids showed significant differences in serum/plasma samples from COPD patients [15]. Sphingolipids levels were associated with different phenotypes of COPD. Sphingomyelins (SM) and glycosphingolipids were related to emphysema and COPD exacerbations, respectively. Integrated approaches of metabolomics and transcriptomics were also applied to explore biological pathways associated with COPD phenotypes and outcomes [16]. Glycerophospholipid metabolism was related to airflow obstruction and COPD exacerbations; however, sphingolipid metabolism was associated with worse lung function outcomes and severity exacerbation requiring hospitalizations. Another study reported that tryptophan catabolism was more active under acute exacerbation of COPD (AECOPD) status compared to stable COPD [17]. COPD was divided into phenotype E and phenotype M by high-resolution computed tomography scanning. Different phenotypes displayed different metabolomic features toward drug treatment, such as the difference of amino acids and carbohydrate metabolism [18]. Moreover, COPD patients with phenotype M had a more significant response to bronchodilator and corticosteroid treatment [2], while phenotype E was more sensitive to anticholinergic treatment [18]. Therefore, metabolomics might have the potential to distinguish different COPD phenotypes and give insights into the therapeutic evaluation of COPD.

The levels of sphingosine, sphingosine 1-phosphate, and lysophospholipids, biomarkers of lung tissue regeneration [19], were normalized after liver growth factor treatment in emphysema patients [20]. Another metabolomic study indicated doxycycline coupled with standard therapy might improve inflammatory response by downregulating the levels of fatty acids and lactate in COPD patients more significantly, compared to standard treatment alone [21].

Pulmonary rehabilitation, an important therapeutic measure, can effectively improve the functional ability and elevate life quality of COPD patients. Recently, more attention has been paid to the effects of nutritional supplementation. Researchers have found that compared with the combination treatment of long-term oxygen therapy (LTOT) and placebo, LTOT coupled with nutritional supplementation of CoQ10 and creatine could markedly improve the lung function and symptoms of COPD patients [22]. Metabolites associated with glycerophospholipid and amino acid metabolism had significant differences between the two groups. In addition, plasma metabolic profiling displayed potential differences in healthy individuals and COPD patients after exercise training. It was noted that the level of lactate showed a significant post-training fall in COPD patients; however, it was increased in healthy subjects, which showed that plasma metabolic profiling might contribute to the phenotypic characterization of COPD patients [23].

#### 2.1.2. Diagnosis of COPD

In general, phosphatidylcholines (PCs) concentrations were reduced in COPD subjects; however, the levels of LysoPCs displayed an increased tendency [4]. Proteostasis imbalance can cause inflammatory response, oxidative stress, and apoptosis [24], which may contribute to the progression of COPD. Several amino acids, such as glutamine and arginine in serum, the degradation markers of proteins, were identified and used for COPD diagnosis with quantitative metabolomics [6,25]. In addition, amino acid, purine, lipid, fatty acid, and steroid metabolism showed significant differences in chronic bronchitis (CB) individuals exposed to tobacco smoke [26]. Similarly, another study indicated that amino acid and lipid metabolism perturbation might cause the occurrence of CB [3] by damaging lung function and increasing the exacerbation rate and mortality of COPD [27]. Moreover, the perturbations of amino acid and lipid metabolism were also observed in HIV-associated COPD patients [28]. In addition, sex-associated oxidative stress and autotaxin–lysophosphatidic acid axis disorder might significantly influence COPD severity [29].

Blood samples are also utilized in metabolomic analysis. Researchers found that carnitine and phenylalanine to tyrosine ratios were associated with increased age in COPD patients [30]. Another study from this team also found BCAAs metabolism and glycolysis dysregulation in serum samples could be adjusted by dexamethasone and bergenin treatment [10].

#### 2.1.3. Differential Diagnosis of COPD

Some metabolites, such as myoinositol, fumarate, and cysteinesulfonic acid, showed significant differences in serum samples from COPD smokers and healthy smokers, which would offer a promising and accessible window for recognition of early-stage COPD [8]. Moreover, different metabolites were related to different clinical parameters. In addition, metabolomics may also help distinguish COPD from other respiratory diseases, for example, lung cancer [11] and acute respiratory failure caused by COPD exacerbation, pneumonia, or heart failure [31]. Compared with COPD, some serum metabolites levels, such as acetate, citrate, and methanol, were decreased in lung cancer, while the levels of leucine, lysine, mannose, and choline were increased.

### 2.2. Urine Sample

Urine is also a common and accessible sample in metabolomics. Several candidate biomarkers in urine might explain the mechanisms of COPD and be beneficial to COPD diagnosis [9]. Stable COPD and acute respiratory failure caused by COPD exacerbation, pneumonia, or heart failure might also be distinguished by some distinct metabolites, such as 3-hydroxymandelate and nicotinamide in urine samples [31]. Asthma–COPD overlap (ACO) manifests a persistent airflow limitation similar to COPD, which combines the features of asthma and COPD [32]. Recently, a metabolomic study revealed that L-histidine in urine might be a specific diagnostic biomarker of ACO [33]. Particulate matter 2.5 (PM2.5) exposure might aggravate metabolic dysfunctions by damaging antioxidation capacity, such as abnormal histidine levels, and influencing energy generation, such as glucose metabolism dysregulation in lung, which were related to lung function decline in COPD patients [34].

### 2.3. EBC

EBC, containing a number of organic compounds [35], was also applied in a respiratory illness study based on metabolomic analysis [36]. The noninvasive and convenient collection method of EBC is easily accepted by volunteers and patients.

α1 antitrypsin deficiency is a genetic factor for COPD [37]. Several metabolites mainly involved in pyruvate metabolism were detected accumulating in EBC samples from the deficient patients in contrast to healthy individuals [38], which were largely the same as those metabolites identified in COPD patients with normal α1 antitrypsin [11,39]. These findings might provide a rationale for reasoning on the role of these molecules as possible biomarkers of pulmonary illnesses. Similarly, another study found the levels of acetone and indole showed diverse changing trend in exhaled breath from COPD patients and healthy controls who smoked or did not smoke [40].

Metabolomic profile had significant differences between COPD and pulmonary Langerhans cell histiocytosis (PLCH), an interstitial lung disease [41]. An inverse behavior of 2-propanol and isobutyrate characterized COPD with respect to PLCH (high/low in COPD, low/high in PLCH). However, acetate and 1-methylimidazole in EBC had similar altered trend between the two groups compared with healthy subjects. Clinical differential diagnosis of asthma and COPD remains a major challenge, especially for individuals who smoke [42]. Metabolomic analysis has been applied to diagnose asthma or COPD in recent years [43]. The levels of ethanol and methanol were higher in COPD than asthma. However, formate and acetone/acetoin ratio showed significantly lower levels in COPD compared with asthma.

A study evaluating inhaled corticosteroids (ICS) efficacy and withdrawal response in COPD patients using metabolomics showed that several metabolites levels, such as formate and acetate, presented evident differences before and after therapy [44].

### 2.4. Lung Tissue Sample

Metabolomic analysis of underlying tissue samples may reveal slight changes of biochemical profile prior to histopathology [45]. An in vivo study found energy production pathways in lung tissue disturbed after whole-CS exposure [46]. Another metabolomics study indicated that the disturbance of lipid metabolism in lung tissue was associated with the occurrence and development of COPD to a large extent, which could be improved by aminophylline therapy [47]. The changes of metabolic pathways in COPD might provide insights and guidance into its pathogenesis.

Metabolomics was also applied to investigate pharmacological action of traditional Chinese medicine. Bufei Yishen formula (BYF) could improve metabolomic dysfunction in COPD rat lungs. Moreover, more attention has been paid to arachidonic acid (AA) metabolism in targeted therapy of COPD [48]. Another study showed Bufei Jianpi formula played a modulating role in lipid metabolism, inflammatory response, oxidative stress, and focal adhesion pathways in COPD patients [5]. Moreover, metabolic pathways enrichment analysis indicated that AA, linoleic acid (LA), glutathione, and glycerophospholipid metabolism were evidently changed. In their previous research, BYF displayed similar effects [49].

### 2.5. Other Samples

The peptide concentrations in BALF samples from COPD patients were evidently related to airflow obstruction and decline of pulmonary function [50]. Metabolomics analysis based on sputum specimens indicated that the disturbance of polyunsaturated fatty acids (PUFAs) metabolism was related to the progression of COPD [7]. Moreover, PUFAs metabolism was apparently increased in AECOPD patients in contrast to stable COPD patients. In addition, it would be interesting and worthwhile to explore the effect of e-cigarettes on the metabolic profile of biological systems. Respiratory toxicants like acrolein in cigarette smoke have been found in e-cigarette vapor [51]. Currently, the influence of e-cigarette liquid on the metabolome of human bronchial epithelial cells has been reported. It significantly increases the levels of arginine, histidine, and xanthine, which indicates that e-cigarettes are not completely harmless and might be a risk factor for COPD [52]. These potential COPD-associated biomarkers could be considered as indicators of normal biological and pathogenic processes, or pharmacological responses to therapeutic intervention.

### 2.6. Metabolic Pathway Analysis

**Figure 1:**
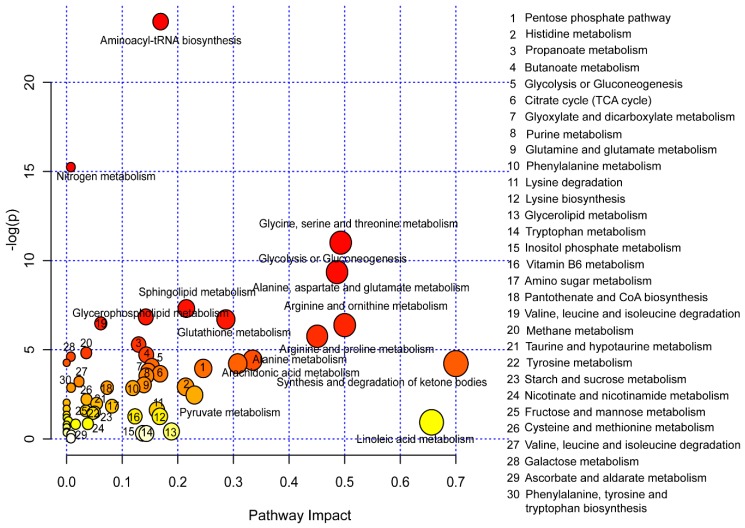
Metabolic pathways analysis based on distinct metabolites published in chronic obstructive pulmonary disease (COPD)-associated metabolomics studies performed by applying the Metabo-Analyst 4.0 platform. The names of 44 disturbed metabolic pathways were marked in the pathway figure, which mainly involved dysfunctions of amino acid metabolism, lipid metabolism, energy production pathways, and imbalance of oxidation and antioxidation.

In an attempt to define relationships among metabolites published in COPD-associated metabolomics studies, a pathway analysis was performed by applying the Metabo-Analyst 4.0 [53] platform and using human as the model organism (Figure 1). Forty-four disturbed metabolic pathways were marked and these metabolic pathways showed evident changes in COPD according to impact >0.1 and *P* < 0.05, mainly involved in the dysfunctions of amino acid metabolism, lipid metabolism, energy production pathways, and imbalance of oxidation and antioxidation.

## 3. The Role of Metabolism Dysfunction in COPD Pathogenesis

**Figure 2:**
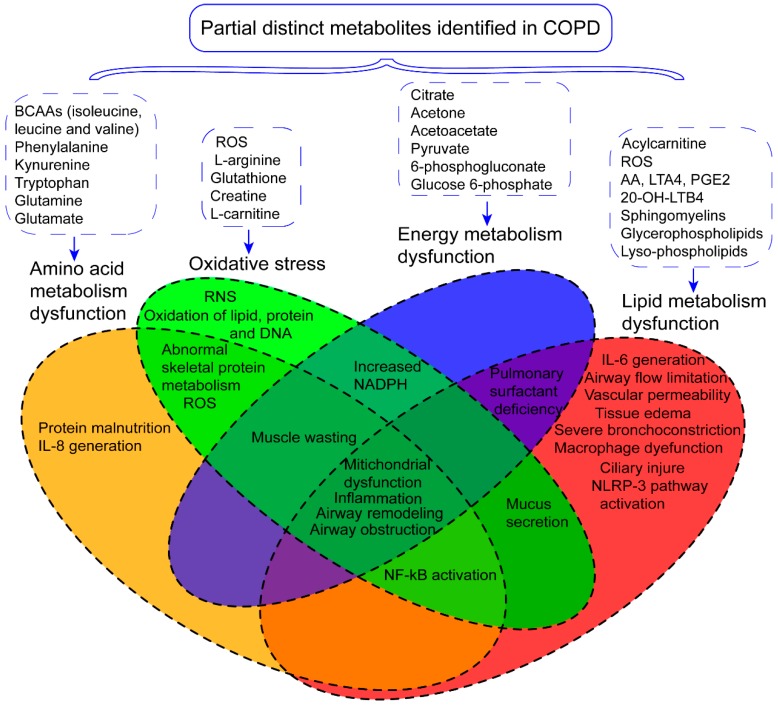
Distinct metabolites identified in COPD-associated metabolomics studies might be involved in the dysfunctions of amino acid metabolism, energy metabolism, lipid metabolism, and imbalance of oxidation and antioxidation, which further contributes to corresponding symptoms and pathology changes. Partial distinct metabolites identified in COPD are shown in the figure. IL-8: interleutin-8; RNS: reactive nitric species; ROS: reactive oxygen species; NF-κB: nuclear factor kappa-light-chain-enhancer of activated B cells; NADPH: nicotinamide adenine dinucleotide phosphate; IL-6: interleutin-6; NLRP3: leucine-rich repeat pyrin 3 domain.

**Figure 3:**
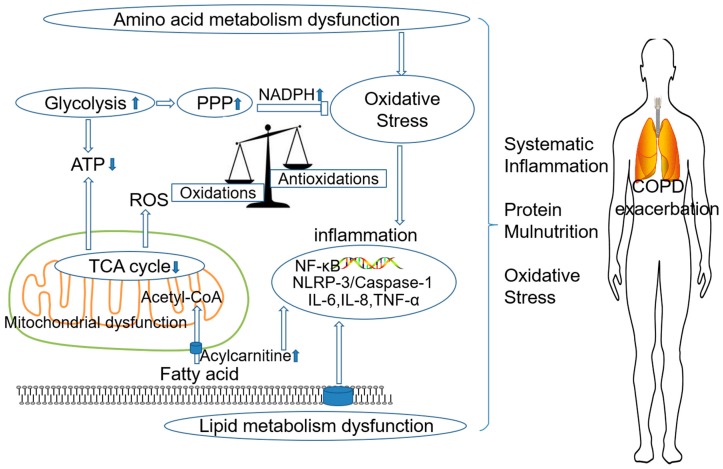
COPD pathogenesis based on metabolomic analysis. The dysfunctions of amino acid metabolism, lipid metabolism, energy metabolism, and imbalance of oxidation and antioxidation cause systematic inflammation, protein malnutrition, and oxidative stress, which may contribute to the development and progression of COPD. ATP: adenosine triphosphate; ROS: reactive oxygen species; TCA: tricarboxylic acid; PPP: pentose phosphate pathway; NADPH: nicotinamide adenine dinucleotide phosphate; NF-κB: nuclear factor kappa-light-chain-enhancer of activated B cells; NLRP3: leucine-rich repeat pyrin 3 domain; IL-6: interleutin-6; IL-8: interleutin-8; TNF-α: tumor necrosis factor-α.

COPD is often affected by multiple genetic and environmental factors [54], yet its exact pathogenesis remains unclear [55]. Herein, we further elucidated the pathological changes (Figure 2) and pathogenesis of COPD according to differential metabolites and disturbed metabolic pathways (Figure 3).

### 3.1. Amino Acid Metabolism Dysfunction and COPD

Anabolism and catabolism of amino acids are related to systematic nutritional status, inflammatory response, and oxidative stress [56]. Weight loss, especially fat free mass loss, is a crucial clinical manifestation of COPD, indicating an active metabolic state in COPD patients [57].

Skeletal muscle is the main protein source under special conditions [58]. BCAAs, including isoleucine, leucine, and valine, can promote protein anabolism and maintain glucose homeostasis in skeletal muscle [59]. Reduced BCAAs levels in COPD may indicate a risk of protein malnutrition. In underweight COPD patients, hypermetabolism caused by COPD exacerbation and respiratory muscle weakness is a main reason for the reduced concentrations of BCAAs [60]. In fact, BCAAs supplement can promote protein synthesis in the elders among COPD patients [61].

Phenylalanine level, reflecting synthesis and breakdown state of the systematic protein, also decreases in COPD. However, phenylalanine concentration has an elevated tendency in GOLD stage IV patients, indicating its level may be related to the severity of COPD [6]. A research founded that high phenylalanine level was evidently related to systemic inflammation [62]. Continuous decrease of indoleamine 2,3-dioxygenase (IDO) activity and the imbalance between interleutin-10 (IL-10) and IL-17A in stable COPD may cause neutrophilic inflammation. However, activity of tryptophan catabolism and its metabolic enzyme, IDO, are increased in AECOPD [63]. Kynurenine, tryptophan’s metabolite, might alleviate inflammatory response by inhibiting the accumulation of Th1 and Th17 cells at the inflammatory site [64]. The reverse results may be a protective mechanism of the biological system under AECOPD conditions. Moreover, tryptophan catabolism may be considered as a special indicator in AECOPD patients.

Glutamine and glutamate also have critical regulatory effects on antioxidative stress and biosynthesis under metabolic dysfunction conditions, such as decreasing glutathione level and negatively regulating nitrogen balance [65]. Glutamine mainly comes from the metabolism of skeletal muscle; however, the lung also has potential to release glutamine under stress conditions, indicating an increased utilization of glutamine in lung tissue [66]. In comparison, glutamine levels increase in COPD, which may be associated with abnormal skeletal muscle protein metabolism and oxidative stress.

### 3.2. Lipid Metabolism Dysfunction and COPD

Abnormal lipid metabolism in COPD may be related to reduced dietary intake and increased resting energy expenditure caused by growing workload of respiratory muscles and anoxia [67]. COPD-associated lipids metabolism disturbance was mainly involved in fatty acids and phospholipids metabolism.

Acylcarnitine, an intermediate of fatty acid β-oxidation (FAO), displayed an increased tendency in COPD [30], which might reflect the growing energy demand from lipid oxidation. Moreover, acylcarnitine can activate inflammatory signal pathways via inducing IL-8 production and nuclear factor kappa-light-chain-enhancer of activated B cells (NF-κB) activation [68]. Acetyl-CoA, a metabolite of FAO, is an essential substrate for the tricarboxylic acid (TCA) cycle, thereby producing adenosine triphosphate (ATP) and reactive oxygen species (ROS) [69]. Moreover, it is also a raw material for synthesis of ketone bodies in mitochondria [70]. In healthy individuals, the levels of blood ketone bodies are low and only increase in the case of increased fat utilization [71]. Therefore, the increased ketone bodies levels in COPD might be related to a shift from carbohydrate oxidation towards lipids usage.

Phospholipids, important components of biological membranes and pulmonary surfactants, can help maintain the membrane fluidity and integrality as well as reduce alveolar surface tension [72,73]. Phospholipid-derived sphingomyelins and glycerophospholipids metabolism are disturbed in COPD. Ceramide, an intermediate product of sphingomyelin metabolism, abnormally accumulates in lung tissue and may damage endothelial-defense, induce alveolar epithelial cell apoptosis, promote inflammatory response, and cause macrophage dysfunction [74]. These pathological features may be associated with the activation of NF-κB and nucleotide binding domain and leucine-rich repeat pyrin 3 domain/caspase-1 [75]. The main metabolites of glycerophospholipids are lyso-phospholipids and PUFAs. Lyso-phospholipids can cause dysfunction of vascular endothelial cells by activating NF-κB and promoting inflammatory cytokine production from leukocytes [76]. In addition, increased oxidative stress in COPD can promote the oxidation of phospholipids and activate the innate immune system, resulting in persistent inflammation [77].

PUFAs metabolism include AA, LA, and alpha-linoleic acid (ALA) metabolism. The levels of metabolites of AA, leukotriene A4 (LTA4), prostaglandin E2 (PGE2), 20-OH-LTB4, and 5-hydroxy-eicosatetraenoic acid, significantly increased in COPD [7]. PGE2 could cause respiratory inflammation by inducing IL-6 generation [78]. In addition, PGE2 can aggravate airflow limitation in COPD, which is associated with increased matrix metalloproteinases expression [79]. AA-derived LTs are related to many lung pathological processes in COPD, such as mucus secretion, inflammatory cell infiltration, vascular permeability change and tissue edema, ciliary injury, as well as severe bronchoconstriction [80]. Free radical-catalyzed AA peroxidations may cause membrane lipid damage in lung, which might also contribute to the progression of COPD [81]. Recent studies have found omega-3 PUFAs, including ALA, eicosapentaenoic acid, and docosahexaenoic acid, play an important role in improving inflammation [82]. The levels of LTB4, tumor necrosis factor-α (TNF-α), and IL-8 are significantly reduced in COPD patients with long-term omega-3 PUFAs supplementation [83]. The concentrations of lipid mediators reducing inflammation are low in COPD, possibly due to their restrained precursor production [7].

### 3.3. Energy Metabolism Dysfunction and COPD

Weight loss, characterized by muscle wasting in COPD, is accompanied by decreased physical performance and respiratory muscle function. Elevated energy metabolism due to nutritional supplementation significantly improves COPD symptoms [84]. Metabolic pathways correlated with energy production, such as glycolysis, pentose phosphate pathway (PPP), and the TCA cycle, are perturbed in COPD according to some metabolomics studies [46].

The imbalance of slow-twitch fibers and fast-twitch fibers in the peripheral skeletal muscle of COPD patients reveals a relative shift from oxidative to glycolytic capacity [85]. Another study suggested that transforming growth factor-β might transfer aerobic oxidation of glucose to glycolysis by modulating mitochondrial ROS levels and mRNA levels of enzymes associated with glycolysis in the airway and cause airway smooth muscle cells hyperplasia, hypertrophy, and airway wall thickening [86]. Also, decreased energy from glycolysis may influence the production of pulmonary surfactant [87].

Mitochondrial dysfunction in lung tissue may cause lung inflammation and airway remodeling [88]. It also drives an increase of glycolysis in COPD. Metabolites of glycolysis are fed into PPP to generate nicotinamide adenine dinucleotide phosphate, which is employed for fatty acid biosynthesis and antioxidant protection [89]. Metabolomic analysis found that PPP was activated in COPD patients with CS exposure [90], which might be a protective mechanism to alleviate oxidative stress. In addition, the abnormal citrate level in COPD reflects the disturbance of TCA cycle, which might be associated with the decreased activities of enzymes involved in oxidative energy metabolism and mitochondrial dysfunction [91]. Reduced ATP levels derived from TCA cycle in COPD are also a cause of systemic dysfunction.

### 3.4. Oxidative Stress and COPD

Most COPD-associated studies indicate that increased oxidative stress caused by long-term CS exposure contributes to lung dysfunction [92,93] and the destruction of energy metabolism homeostasis [94]. CS exposure may cause leukocyte activation, ROS production [95], and nitric oxide (NO) bioavailability reduction [96]. Diverse endogenous and exogenous factors cause the production of ROS, directly associated with compounds oxidation such as proteins, lipids, carbohydrates, and DNA [92]. Fractional exhaled nitric oxide (FeNO) is viewed as an inflammatory indicator in respiratory diseases. NO, a common and highly reactive free radical in living systems, is converted by arginine oxidation and nitrite reduction [97]. Metabolomic data of COPD also shows an increase of arginine level in COPD. L-arginine needs NO synthase and arginase to synthesize NO and its downstream amino acid. A study indicated that L-arginine catabolism transformed from the NO synthase pathway to arginase pathway in COPD, contributing to airway obstruction and chronical airway remodeling [97]. However, a possible reason for the high FeNO concentration in COPD is the decrease of NO bioavailability, which may cause nitrite accumulation. Nitrite can induce the production of reactive nitrogen intermediates and aggravate oxidative stress [98].

Glutathione (GSH), an antioxidant in the epithelial lining fluid [99], is an essential intermediate in the conversion of hydrogen peroxide to water and lipid peroxides degradation [100]. In addition, GSH might improve inflammation by inhibiting NF-κB activation. Metabolomic studies have revealed altered GSH level, which might be associated with the increased oxidant concentrations in lung tissue of COPD patients [101]. The main function of creatine is to provide phosphate groups for the conversion from adenosine diphosphate to ATP [102]. Several studies also reported that creatine might protect lung function from oxidative stress damage by reducing superoxide anions and peroxynitrite [22]. The β-oxidation process of long chain fatty acids in the mitochondria depends on the transport of a vital antioxidant, L-carnitine [103]. A decreased L-carnitine level in COPD is also related to oxidative stress.

## 4. Conclusions

High-throughput metabolomics studies have widely covered the pathogenesis, phenotypic differentiation, diagnosis, and therapeutic evaluation of COPD. Differential biomarkers in various samples can be identified based on NMR and MS techniques. However, confirmed biomarkers associated with COPD have not yet been reported. Therefore, the sensitivity and specificity of these distinct metabolites need proper validation in large populations and in longitudinal studies [104]. It is difficult to get fully identical results between laboratories due to the differences of equipment and laboratory-specific practices. However, data sharing, method synchrony, and sample storage integrity, might allow high reproducibility and reusability of data from COPD-associated metabolomics [105,106]. It is known that the dysregulation of amino acid metabolism, lipid metabolism, energy production pathway, and oxidative stress are significantly related to the development and progression of COPD by metabolic pathways analysis. However, most studies are currently limited to the identification of differential metabolites and lack the verification process of relevant signaling pathways. Subsequent studies should also focus on the changes of genes and proteins associated with the perturbed metabolic pathways. In addition, multiomic data analysis, integrating metabolomics with proteomics, genomics, epigenomics, transcriptomics, and microbiomics, will help provide deeper insights into the pathological mechanisms of COPD.

## Figures and Tables

**Table 1:** A summary of COPD-associated metabolomic studies.

Author and Year	Subjects	Sample/Platform	Metabolites Name	
			Increase	Decrease
Bowler, R.P. et al. (2015) [15]	129 current and former smokers from the COPD Gene cohort	Plasma/HPLC-MS	Trihexosylceramides, Dihexosylceramides, Sulfatide d18.1. N16.0, Ganglioside GD1.d18.1. N16.0 (COPD exacerbations)	Ceramide, Sphingomyelin, Ganglioside, GM3, Sphingomyelin (Emphysema) Sphingomyelin, S1P (COPD exacerbations)
Kilk, K. et al. (2018) [4]	COPD patients (n = 25) and control individuals (n = 21)	Serum/HPLC-MS	LysoPC	SM, Hydroxylated SM, Arginine, Proline
Navarrete, A. et al. (2017) [20]	Control group and CS-exposed group without (n = 6, n = 10) or with (n = 10, n = 8) LGF treatment	Plasma/LC-QTOF-MS	Lysophosphatidylcholines, Mandelic acid, Hydroxymethylbenzoic acid (Before therapy), Sphingosine, Sphingosine 1-phosphate, Lysophospholipids (After LGF therapy)	Phenylalanine, Sphingosine 1-phosphate Sphingosine, Hydroxylysine, Dodecenoic acid, Oxo-methylthiobutanoic acid, (Before therapy)
Brajesh, S. et al. (2017) [21]	COPD patients receiving standard therapy (n = 40) and combination of doxycycline and standard therapy (n = 60)	Serum/NMR	Formate, Citrate, Imidazole, L-arginine (After doxycycline therapy compared with Pre-treatment group)	Lactate, Fatty acid (After doxycycline therapy compared with Pre-treatment group)
Ubhi, B. K. et al. (2012) [6]	Controls (n = 66) and GOLD stage II (n = 70), III (n = 64) and IV (n = 44) COPD patients	Serum/NMR LC-MS/MS	Glutamine, Phenylalanine, 3-methylhistidine, Ketone bodies	Lipoproteins, BCAAs, Glycine, Creatine, N, N-dimethylglycine
Ubhi, B. K. et al. (2012) [25]	GOLD IV patients (n = 30) and controls (n = 30)	Serum/LC-MS/MS	Glutamine, Aspartate, Arginine	Aminoadipate
Novotna, B. et al. (2018) [30]	COPD patients (n = 10) and healthy controls (n = 10)	Blood/HPLC-MS/MS	Carnitine, Phenylalanine/Tyrosine	Alanine, Phenylalanine, Pyroglutamate Free Carnitine/Acylcarnitine
Ren, X. et al. (2016) [10]	SD male rats include control, model, dexamethasone and bergenin groups, with 10 rats in each group	Serum/NMR	Isobutyrate, Acetone, Acetoacetate, Pyruvate, Glycine, Glycerol, Threonine (Before therapy). Glutamine, Glucose (After dexamethasone therapy). Glutamine (After bergenin therapy)	Isoleucine, Leucine, Valine, Lactate, Alanine, Proline, Glutamine, Glutamate, Creatine phosphate, Glucose, Serine (Before therapy). Pyruvate, Glycine, Threonine (After dexamethasone therapy). Pyruvate, Glycine, Threonine (After bergenin therapy)
Cruickshank-Quinn, C. I. et al. (2014) [26]	DBA/2J mice included controls group (n = 3), CS- exposed group for different time	Plasma/LC-MS	Homocitrulline, Arginine, Phenylacetylglycine, PI (36:2), PS (28:2), TG, Adenosine, AMP, Hypoxanthine	Glycerophospholipids, Glycerolipids, Pregnanetriol, Pentadecanoylglycine
Ren, X. et al. (2016) [3]	40, 60, and 80% TS groups of rats (n = 6) and control group of rats (n = 6)	Serum/LC-MS	Lysophosphatidylethanolamine, Lysophosphatidic acid (18:1), Docosahexaenoic acid, 5-hydroxyindoleacetic acid, 5′-carboxy-γ-tocopherol	4-imidazolone-5-propionic acid, 12-hydroxyeicosatetraenoic acid, Uridine
Hodgson, S. et al. (2017) [28]	HIV-associated COPD patients (n = 38), controls (n = 38)	Plasma/LC-MS/MS	Kynurenine/Tryptophan ratio, Ceramide, Fatty acids	Diacylglycero
De Benedetto, F. et al. (2018) [22]	90 COPD patients received supplementation with QTer^®^ and Creatine or placebo	Plasma/LC-MS	SM (OH) C16:1, SM C18:0 (supplementation with placebo) Lysophosphatidylcholine (Supplementation with QTer^®^ and Creatine)	Phosphatidylcholine, Sphingomyelins (Supplementation with QTer^®^ and Creatine)
Rodríguez et al. (2011) [23]	the effects of exercise on COPD patients (n = 18), healthy subjects (n = 12)	Plasma/NMR	glutamine, tyrosine, alanine, valine and isoleucine, creatine, creatinine, citrate and glucose (healthy subjects after training)	lactate, succinate and pyruvate (healthy subjects after training) lactate (COPD patients after training)
Wang, C. et al. (2017) [2]	Stable COPD patients with phenotype E (n = 22) and phenotype M (n = 28)	Serum/NMR	ADP, Guanosine, Choline, Glycine, Proline, Tyrosine, L-alanine, L-valine, Leucine (E and M) L-threonine, (E), Malonate (M)	Acetone, Lactate (E and M) Pyruvic acid(E), Uridine(M)
Tan, L.C. et al. (2018) [18]	COPD patients with phenotype E (n = 20) and phenotype M (n = 24)	Serum/NMR	Fructose, Glycine, Pyruvic acid, Pyruvate, Proline, Acetone, Lipid CH2CH2CO, Threonine, Lsopropyl alcohol (E and M), Lactate, Creatine, Citric acid, L-glutamine, Maltose Ornithine, 2-hydroxyisobutyrate, L-threonine, L-valine, Glutamic acid, β-alanine, Betaine, Cyclopentane (E); N-acetylcysteine (M)	Asparagine, pyridoxine(E) Ornithine, Guanosine, Lipoprotein(M)
Chen, Q. et al. (2015) [8]	Healthy smokers (n = 37), COPD smokers (n = 41) and non-smokers (n = 37)	Serum/LC-MS	Fibrinogen peptide B, Myoinositol, Dimethyluric acid, N-methylnicotinate, Cysteinsulfonic acid, Glycerophosphoinositol, Phosphatidylinositol (40:7), Creatinine	Several hydrophobic unknowns (with chromatographic retention time consistent with fatty acids and lipids)
Deja, S. et al. (2014) [11]	COPD patients (n = 22) and lung cancer (TNM stages I, II, III, and IV) patients (n = 77)	Serum/NMR	N-acetylated, Glycoproteins, Leucine, Lysine, Mannose, Choline, Lipid (CH3 (CH2) n) (lung cancer compared with COPD)	Acetate, Citrate, Methanol (lung cancer compared with COPD)
Fortis, S. et al. (2017) [31]	Stable COPD patients and acute respiratory failure patients caused by COPD exacerbation, pneumonia or heart failure	Serum Urine/NMR	Glutamine, Formate, Alanine, Proline, Histidine, Creatine, Phosphate (Serum); Cis-aconitate, Oxoglutarate (Urine, stable COPD compared with acute respiratory failure)	Mannitol, Citrate (Serum); Furoylglycine, N-oxide, Methyl-2-oxovalerate, Niacinamide Nicotinamide, (Urine, stable COPD compared with acute respiratory failure)
Naz, S. et al. (2017) [29]	Healthy, smokers and smokers with COPD	Serum/LC–MS	Asymmetric, Asymmetric/symmetric dimethylarginine, Fatty acid, Sphingolipid pathways (female); cAMP signaling (male)	Acetyl–ornithine/Ornithine, Arginine/(citrulline+ornithine) (female)
Wang, L. et al. (2013) [9]	COPD patients (n = 32) and healthy controls (n = 21)	Urine/NMR	Acetate, Ketone bodies, Pyruvate, Carnosine, M-hydroxyphenylacetate, Phenylacetyglycine,	1-methylnicotinamide, Creatinine, Lactate
Huang, Q. et al. (2018) [34]	41 Chinese elderly participants including COPD patients and their healthy spouses	Urine/ICP-MS	N-formyl-l-methionine, CPA, Decanoylcarnitine, L-histidine, Spermine, Acetylcarnosine, L-octanoylcarnitine, D-glucose (COPD) Indolelactic acid, 5-phosphoribosylamine (PM2.5 exposure)	Suberylglycine, 3-dehydrocarnitine (COPD), Uric acid, Glyceric acid, 1,3-biphosphate, Methyluric acid, 4-pyridoxic acid, Dopamine 4-sulfate, (PM2.5 exposure)
Airoldi, C. et al. (2016) [38]	ZZ-α1-antitrypsin deficient COPD patients (n = 11) and healthy controls (n = 11)	EBC/NMR	Acetate, 2,3-butanediol propionic acid, Lactate, Butyrate acetone, Benzoate, fatty acid, Formate, Alanine, Ethanol, Acetoin, Isopropanol acetoin, Propionate, Acetate	No report
Laurentiis, G. et al. (2013) [41]	Healthy smokers (n = 20), COPD smokers (n = 15), PLCH patients (n = 15)	EBC/NMR	Acetate (COPD and PLCH) 2-propanol (COPD) Isobutyrate (PLCH)	1-methylimidazole (COPD and PLCH) Isobutyrate (COPD) 2-propanol (PLCH)
Ishikawa, S. et al. (2019) [46]	3D bronchial tissue	3D bronchial tissues/LC-MS/MS	6-phosphogluconate, Erythrose 4-phosphate Ribose 5phosphate (R5P)	Glucose 6-phosphate, Fructose 6-phosphate, Glutathione, Oxidized glutathione, ADP, ATP
Li, J. et al. (2017) [47]	COPD rats treated with normal saline (n = 10) and aminophylline (APL) (n = 10)	Lung tissue/LC-QTOF/MS	LTA4, 5-HETE, 20-OH-LTB4, LXA4, PGE2 (Before ALP therapy)	LTA4, 5-HETE, LTB4, LXA4, PGE2 (After ALP therapy)
Yang, L. et al. (2015) [48]	SD rats included control group (n = 40), COPD group (n = 40), and BYF therapy group (n = 40)	Lung tissue/HPLC-Q-TOF/MS	Linoleic acid, Acetylcholine, Arachidonic acid, 2-methoxyestradiol 20-hydroxy-PGE2, 5-HEPE, 7-Oxo-11-dodecenoic acid, Acetyl-l-leucine (Before BYF therapy) Phenylpyruvic acid, Sphinganine PC (18:1) (After BYF therapy)	Phenylpyruvic acid, Sphinganine PC (18:1) (Before BYF therapy) Linoleic acid, Acetylcholine, Arachidonic acid, 2-methoxyestradiol 20-hydroxy-PGE2, 5-HEPE, 7-Oxo-11-dodecenoic acid, Acetyl-l-leucine (After BYF therapy)
Zhao, P. et al. (2017) [5]	COPD rats treated with normal saline, BJF, and aminophylline	Lung tissue/LC-MS	Glutathione (After BJF therapy)	Arachidonic acid, Linoleic acid, Glycerophospholipid (After BJF therapy)
van der Doesa, A. M. (2018) [7]	Smoking controls, COPD patients in a stable or acute exacerbation phase	Sputum/LC-MS	No report	ALA, EPA, LA, HEPEs, HDHAs, HETEs, LTB4
